# Development of a methodology for in vivo follow-up of hepatocellular carcinoma in hepatocyte specific *Trim24*-null mice treated with myo-inositol trispyrophosphate

**DOI:** 10.1186/s13046-016-0434-8

**Published:** 2016-09-29

**Authors:** Mihaela Ignat, Cherif Youssef Akladios, Véronique Lindner, Konstantin Khetchoumian, Marius Teletin, Didier Muttter, Pierre Marc Aprahamian, Jacques Marescaux

**Affiliations:** 1 IRCAD, 1 place de l’hôpital, 67091 Strasbourg, France; 2 Department of Digestive and Endocrine Surgery, University Hospital of Strasbourg, 1 place de l’Hôpital, 67091 Strasbourg, France; 3 Institute of Genetics and Molecular and Cellular Biology, F-67404 Illkirch, France; 4 Laboratoire de génétique moléculaire, Institut de recherches cliniques de Montréal (IRCM), Montréal, QC H2W 1R7 Canada

**Keywords:** High-resolution micro-computed tomography (micro-CT-scan), Genetically induced hepatocellular carcinoma, Hepatocyte-specific *Trim24*-null mouse, Transgenic mouse, Myo-inositol trispyrophosphate (ITPP)

## Abstract

**Background:**

Genetically induced hepatocellular carcinoma (HCC) models are generally used to investigate carcinogenesis pathways, but very few attempts were made to valorize them for pharmacological testing. This study describes a micro-computed tomography (micro-CT) - based methodology for the diagnostic and lifelong follow-up of HCC in the hepatocyte-specific *Trim24*-null mouse line. Myo-inositol trispyrophosphate (ITPP) was tested as anti-cancer drug.

**Methods:**

Partial hepatectomy was performed in 2 months-old *Trim24*-null mice, in order to accelerate the carcinogenesis process. HCC diagnosis was obtained by micro-CT scan with double contrast agent: 10 μl/g Fenestra™ LC was injected intraperitoneally 6 h prior to imaging and 10 μl/g Fenestra™ VC was injected intravenously 15 min prior to imaging. Twenty three hepatocyte-specific *Trim24*-null mice were considered for ITPP testing (3 mg/g/week intraperitoneally during 10 months in 12 mice, versus 11 controls). Lifelong follow-up was performed using micro-CT. Comparative analysis was performed using unpaired *t* test with Welch correction and survival curves were compared by log-rank test. *Gene expression* analysis was performed using the *RT q-PCR* technique.

**Results:**

Double contrast micro-CT scan allowed HCC diagnosis as hypodense, isodense or hyperdense nodules. Positive predictive value was 81.3 %. Negative predictive value was 83.3 %. Tumor growth could be objectified by micro-CT scan before the ITPP treatment was started, and at 3 and 9 months follow-up. Significant progression of tumor volume was demonstrated in the both groups, with no difference between groups (*p* > 0.05). In the ITPP group, a mild decrease in tumor doubling time was first observed (31.9 +/− 12 days, *p* > 0.05) followed by a significant increase (59.8 +/− 18.3 days, *p* = 0.008). However, tumor doubling time was not different between groups (*p* > 0.05). Median survival after treatment initiation was 223 days (controls) versus 296 days (ITPP group, *p* = 0.0027). HIF1α, VEGF, glutamine synthase, osteopontin expression levels were not significantly modified at the end of follow-up. In the ITPP group, the p53 expression profile was inversed as compared to the control group, higher in non-tumor livers than in tumors.

**Conclusion:**

ITPP treatment allowed for a two-month survival improvement, with better tolerance of tumor burden and apoptosis increase in non-tumor, pathological livers.

**Electronic supplementary material:**

The online version of this article (doi:10.1186/s13046-016-0434-8) contains supplementary material, which is available to authorized users.

## Background

Hepatocellular carcinoma (HCC) is the sixth most common cancer in the world and the second leading cause of cancer mortality [1]. HCCs’ prognosis is poor due to chemo and radiotherapy resistance [2, 3]. Consequently, there is a serious need for new treatment options. Small animal models are often the only available means of testing the safety, potency, and efficacy of new anticancer agents prior to clinical trials. Several types of mouse models of HCC are available, depending on tumor-inducing mechanisms. For drug development, the most frequently used models are xenograft models. They are easy to obtain and follow up, with a rapid growth rate, but their relevance is limited, as the resemblance between xenograft tumors and human HCC is rather poor [4]. Additionally, there are significant differences in tumor growth inhibition between HCC cell lines [5].

Spontaneous developing tumor models (genetically engineered mice) are more relevant as tumors occur through a multistep process of hepatocarcinogenesis. The natural history is respected (hepatocyte proliferation, dysplasia, neoplasia) and HCCs result from the cooperation and dependency between oncogenes, growth factors and viral genes [5]. Genetically induced tumor models are generally used to investigate carcinogenic pathways, but very few attempts were made to use them for pharmacological testing.

A hepatocyte-specific *Trim24*-null mutant mouse line was used in this study. TRIM24 is a ligand-dependent nuclear receptor co-regulator interacting with Retinoic Acid Receptors (RARs). It was shown to function as a potent liver-specific tumor suppressor by attenuating RAR alpha mediated transcription. Indeed, in genetically engineered mice with silencing of *Trim24* gene, an aberrant activation of RAR alpha, leads to sequentially development of hepatocyte alteration, preneoplastic lesions, and HCC [6, 7]. In order to perform pharmacological testing in this spontaneously developing HCC model, it was necessary to assess the presence and to ensure the follow-up of intra-abdominal tumor growth without animal sacrifice.

Micro-computed tomography (micro-CT) for small animal imaging has been increasingly used over the last decade [8]. Liver imaging has been obtained with the enhancement of a radiological agent either through an ApoE receptor-mediated mechanism or a mechanism of nanoparticles uptake by the reticuloendothelial system [9]. Both types of contrast agents resulted in variable quality of liver imaging depending on dose, imaging timing, subjacent liver pathology [10], and even mouse strain [11].

In this study, two main objectives are addressed: first, to provide a reliable imaging method for spontaneously developing liver tumors, and secondly, to evaluate the response to a new anticancer drug, namely myo-inositol trispyrophosphate (ITPP) [12–14] in the hepatocyte-specific *Trim24*-null mutant mouse model.

## Methods

### Animal models

#### Hepatocyte-specific *Trim24*-null mice (*Trim24*^L2/L2^Alb-Cre)

A hepatocyte-specific *Trim24*-null mouse in mixed C57BL/6 x129/Sv mouse strain was used as a spontaneous developing HCC model. As previously described [6], *Trim24*-null mice as well as hepatocyte-specific *Trim24*^L2/L2^Alb-Cre [15] develop sequentially abnormal hypertrophic hepatocytes with enlarged nuclei and increased DNA content and ploidy (3 months of age), clear-cell foci of altered hepatocytes (7 months), adenomas (from 9 months), and invasive hepatocarcinomas (from 12 months) giving rise to lung metastases.

**Wild type mice** were 2 months old male littermates of the *Trim24*^L2/L2^Alb-Cre mice.

### Partial hepatectomy

Based on the principle of partial hepatectomy (PH) as promoter of liver carcinogenesis [16], a two-third partial hepatectomy was performed in *Trim24*^L2/L2^Alb-Cre males at the age of 2 months. Surgery was performed under isoflurane/oxygen general anesthesia (induction at 3 %, maintaining 1.5 %). A right subcostal laparotomy was performed. A single silk thread was used for suture of the left and medial lobes of the liver (constituting approximately 70 % of the total liver mass) along with the gallbladder. Gallbladder, left and medial lobes were resected, followed by careful hemostasis. The abdominal wall was closed with Vicryl 3/0 separate sutures in two layers (muscular layer and skin). Animals were allowed to recover on a 37 °C warm plate.

The early and medium-term effects of PH were evaluated in five groups of 10 mice (5 wild-types and 5 *Trim24*^L2/L2^Alb-Cre). Mice were sacrificed at 2 months of age, and postoperatively at 48 h, 72 h, 5 days, and 15 days respectively after PH. The long-term effect of PH was evaluated in a group of 6 *Trim24*^L2/L2^Alb-Cre mice, which underwent a PH at the age of 2 months and were sacrificed postoperatively at 5 months. Livers were harvested for analysis under isoflurane/oxygen general anesthesia prior to euthanasia.

#### HCC xenograft model

This model was obtained as previously described, by means of an orthotopic injection of a suspension of 2 × 10^6^ Hep 55.1C cells in C57BL/6 J mice [17].

### Micro-CT-scan

A small animal micro-scanner X (micro CAT II - Imtek/Siemens Medical Solutions, Malvern, PA) was used. The micro CAT II scanner has a single X-ray source and detector technology, and it provides reconstructed images with a voxel size of 119x119x119μm. The technical parameters employed were the following: exposure time: 300 milliseconds, X-ray voltage: 80.0 kVp, and anode current: 500 μA. Micro-CT-scan imaging was performed under isoflurane general anesthesia and with respiratory gating. Images were captured after peak expiration, resulting in a mean scanning time of 15 min. Radiological contrast agents Fenestra™ LC and Fenestra™ VC (ART Advanced Research Technologies Inc., Montreal, Canada) consist in polyiodinated lipids, which are selectively taken up by hepatocytes via an ApoE receptor-mediated pathway. Contrast agent injection was performed in conscious mice, placed in a restraining tube and light heated. In order to prevent volume overload and liver toxicity (see Additional file 1), contrast agent dosages, route and timing of administration were established as follows: Fenestra™ LC (10 μl/g) intraperitoneally, 6 h prior to imaging and Fenestra™ VC (10 μl/g) intravenously in the tail vein, 15 min prior to imaging.

Micro-CT-scan images exported by the Amira™ 3D software were processed with 3D-VPM software (IRCAD, Strasbourg, France) for 3D reconstruction and to compute tumor volumes.

To evaluate micro-CT-scan accuracy, a group of 28 *Trim24*^L2/L2^Alb-Cre mice aged between 3 and 25 months were imaged by means of micro-CT-scan, and then dissected and histologically examined.

### HCC follow-up under ITPP treatment - study design

A group of 23 hepatocyte-specific *Trim24*-null mice was considered for this study of HCC follow-up under ITPP treatment (ITPP was a gift from Jean Marie Lehn, ISIS, Strasbourg, France). All mice had partial hepatectomy at 2 months of age and three successive micro-CT assessments, at 5, 12 and 15–16 months of age. Then, ITPP treatment was started in 12 mice using an intraperitoneal injection of 3 mg/g/week over 10 months, and 11 untreated mice were considered as controls. All 23 mice were imaged by means of micro-CT scan at 3 and 9 months after treatment/survey initiation. 3D reconstructions of tumor and liver were performed: tumor volumes were calculated, allowing for the measurement of tumor doubling time (TDT) [18] and growth rate.

Follow-up was performed until mice were terminally ill (cachexia, bad grooming, major weakness) or reached 25 months of age, when the eight surviving mice (5 in the ITPP group and three in the control group) were sacrificed. Dissection, macroscopic measurements and sampling for histology and molecular analysis were performed. Liver and tumor measurements at dissection were corroborated with the last micro-CT-scan rendering.

Survival curve was constructed over 25 months of age (average lifespan of wild-type mice).

Animal experiments were approved by the local ethics committee and were performed according to the revised European Community directive (2010/63/EU, September 24, 2010) for the protection of animals used for scientific purposes.

### Gross examination, histology, and molecular biology methods

*Gross examination* was performed on livers harvested under isoflurane anesthesia. Livers were weighed first. If present, the number of tumors, their measurements and their localization were recorded. Tumor volume was calculated as D1 x D2 X D3 x π/6, where D1, D2 and D3 are tumor diameters.

*Histology* was performed on tissue samples, fixed in buffered formaldehyde for 24 h, dehydrated and paraffin-embedded. Standard 5-μm slides were obtained and stained by routine Hematoxylin - Eosin (HE) staining. Slide analysis was performed using the AxioVision 4.6 software (Carl Zeiss MicroImaging GmbH, Göttingen, Germany).

Ki67 labeling was used for evaluation of proliferation rate and TUNEL assays (Terminal deoxynucleotidyl transferase dUTP nick end labeling) for apoptosis assessment, according to standard procedures. The percentage of Ki67-positive cells and TUNEL-positive cells was determined from five randomly chosen fields/section and three sections/liver for each animal.

#### Molecular biology

*Trim24 genotyping* was performed on genomic DNA obtained from tail biopsy sampled on 10-day-old mice, by means of a standard PCR technique, using Chromo 4 Thermocycler (Bio-rad, Marnes-la-Coquette, France). Primers sequences are available in Additional file 2.

*Gene expression* analysis in tumor and normal liver samples was performed using the *RT q-PCR* technique. Tissue samples were homogenized in a FastPrep®-24 Instrument (MP Biomedicals Inc., lllkirch, France) in Lysis Matrix B tubes (MP Biomedicals, Inc.) containing lysis buffer (Sigma-Aldrich, Saint Quentin Fallavier, France). RNAs were then purified with the mammalian GenElute Gel Extraction Kit (Sigma-Aldrich), according to the manufacturer’s recommendations. RNAs (3 μg) were used as template for reverse transcription with random hexamer and anchored oligo dT, in the presence of 200 units of reverse transcriptase (MP Biomedicals, Inc.). Resulting cDNAs were analyzed using the RT qPCR on a Chromo 4 Cycler, using QuanTitect MasterMix (Qiagen, Courtaboeuf, France) and the primers corresponding to genes of interest (see Additional file 2). Results were analyzed with the Opticom 3 software (Bio-rad). Expressions of genes of interest were normalized by housekeeping gene HPRT and represented as tumor/liver ratio.

### Statistical analysis

Data are expressed as mean ± standard deviation. Comparative analysis was performed using a two-way analysis of variance and unpaired *t* test with Welch correction (*p* < 0.05 was considered statistically significant). Linear regression equations, confidence intervals, and log-rank tests were performed using the GraphPad InStat statistics software (GraphPad Software, Inc., La Jolla, CA). Correlation between data series was evaluated using Pearson or Spearmen correlation coefficient as appropriate. The determination coefficient R^2^ < 0.5 was interpreted as an absence of correlation, and values close to one as a strong correlation.

## Results

### Acceleration of hepatocarcinogenesis process by means of partial hepatectomy (PH)

Early and medium-term consequences of PH were evaluated by relative liver mass measurements, proliferation and apoptosis levels. Relative liver mass measurements showed an impaired liver regeneration in *Trim24*^L2/L2^Alb-Cre mice, with 63.3 % of liver mass recovered at 72 h, versus 73.8 % in wild types. The difference was statistically significant on the overall 15 days period (two tailed student test, *p* = 0.034).

**Fig. 1:**
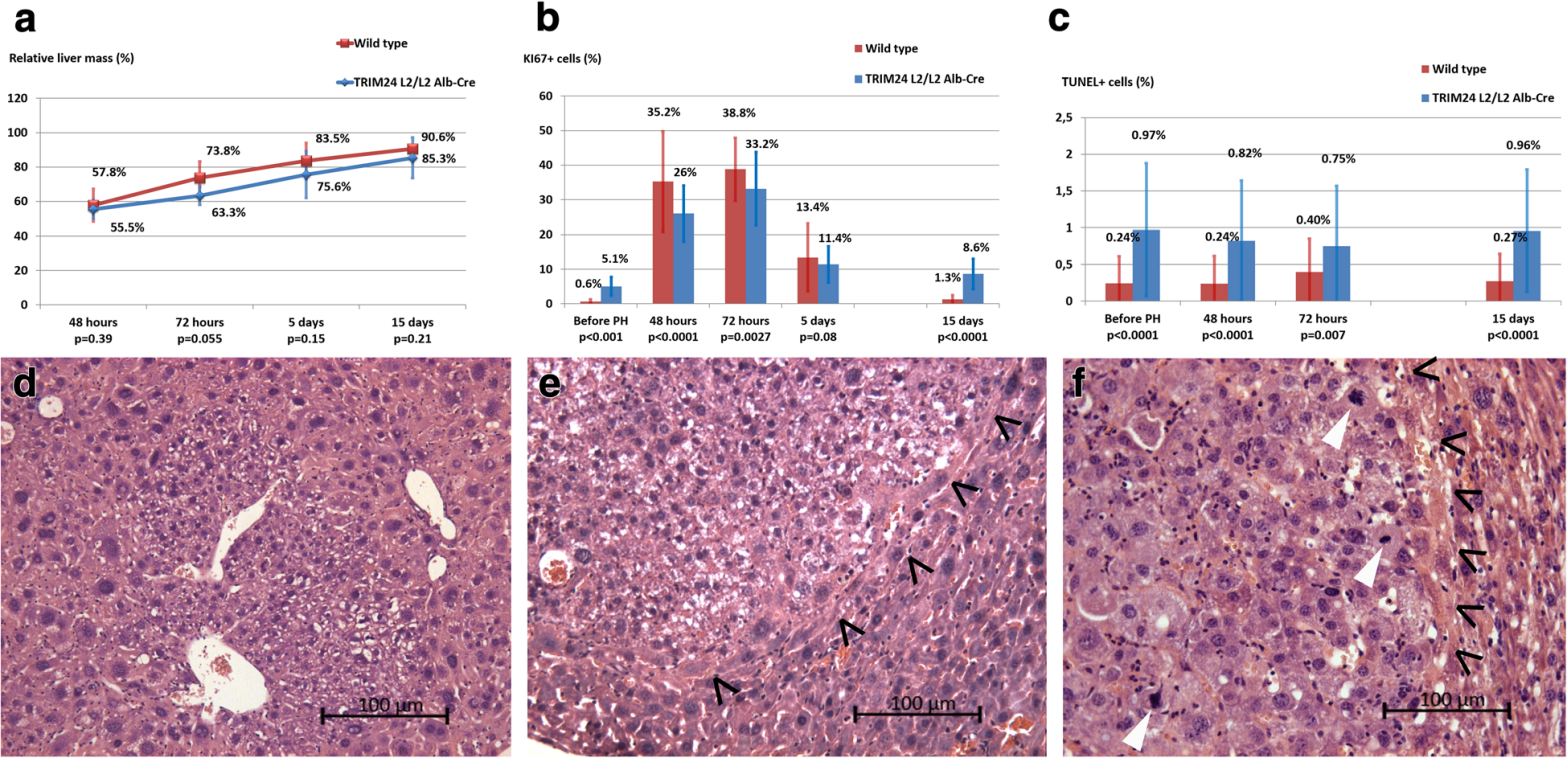
Early and late effects of partial hepatectomy (PH) in *Trim24* ^L2/L2^Alb-Cre mice. **a**, **b**, **c** Early effects of PH in *Trim24* ^L2/L2^Alb-Cre mice versus wild type littermates. **a** Relative liver mass showed impaired and delayed liver regeneration, persistent during at least 15 days in *Trim24* ^L2/L2^Alb-Cre groups. Relative liver mass was obtained as following: mean liver mass of mice at the age of 2 months was measured in the first group (5 mice *Trim24* ^L2/L2^Alb-Cre and five mice wild type). In the groups sacrificed respectively at 48 h, 72 h, 5 and 15 days, the harvested livers were weighed and the relative liver mass was calculated as a percentage of the mean liver mass at the age of 2 months. **b** Ki67 labeling showed significantly lower proliferation rates at 48 and 72 h after PH with abnormal prolonged proliferation at 15 days after surgery in *Trim24* ^L2/L2^Alb-Cre mice. **c** Apoptosis level (TUNEL labeling) demonstrated a significantly higher level of apoptosis in *Trim24* ^L2/L2^Alb-Cre mice, not influenced by the realization of PH. **d**, **e**, **f** Late effect of PH: histological findings (HE staining, original magnification x240) in the livers of *Trim24* ^L2/L2^Alb-Cre mice at 5 months after PH. **d** Clear cell foci (dysplastic hepatocytes, with preserved lobular structure of the liver and no compression) in 5 out of 6 *Trim24* ^L2/L2^Alb-Cre mice. **e** Hepatic adenoma in 3 out of 6 *Trim24* ^L2/L2^Alb-Cre mice, with no portal structure within the affected area and compression of neighbouring hepatocyte plates (black arrow heads). **f** Invasive hepatocarcinoma in 1 out of 6 *Trim24* ^L2/L2^Alb-Cre mice, with loss of portal structures, carcinoma infiltration of neighbouring hepatocyte plates (black arrowheads) and numerous mitotic figures as hallmark of malignant lesion (white arrowheads)

Proliferation rate assessment by Ki67 labeling showed a lower exponential cell cycle entrance at 48 h and lower proliferation peaks at 72 h in *Trim24*^L2/L2^Alb-Cre mice compared to wild types (*p* < 0.001 and *p* = 0.0027 respectively; Fig. 1b). Fifteen days after PH, the proliferation rate was 8.6 % in *Trim24*^L2/L2^Alb-Cre livers, in favor of prolonged proliferation, as compared to the near quiescence state (1.3 %) achieved in wild type mice (Fig. 1b).

Apoptosis level assessment by TUNEL-positive cell rate was higher in *Trim24*^L2/L2^Alb-Cre livers compared to wild type littermates (*p* < 0.01, Fig. 1c). Apoptosis level wasn’t altered following PH in any of the groups (*p* > 0.05 compared to baseline, respectively, Fig. 1c).

Long term effect of PH was evaluated 5 months after surgery. Histology showed the presence of pre-tumor and tumor lesions: clear cell foci (83 %), adenoma (50 %) and invasive hepatocarcinoma (16.7 %), compared to 13 %, 0 % and 0 % in non-operated mice [6] (Fig. 1d, e, f).

### Contrast-enhanced micro-CT-scan imaging of spontaneously developing liver tumors

**Fig. 2:**
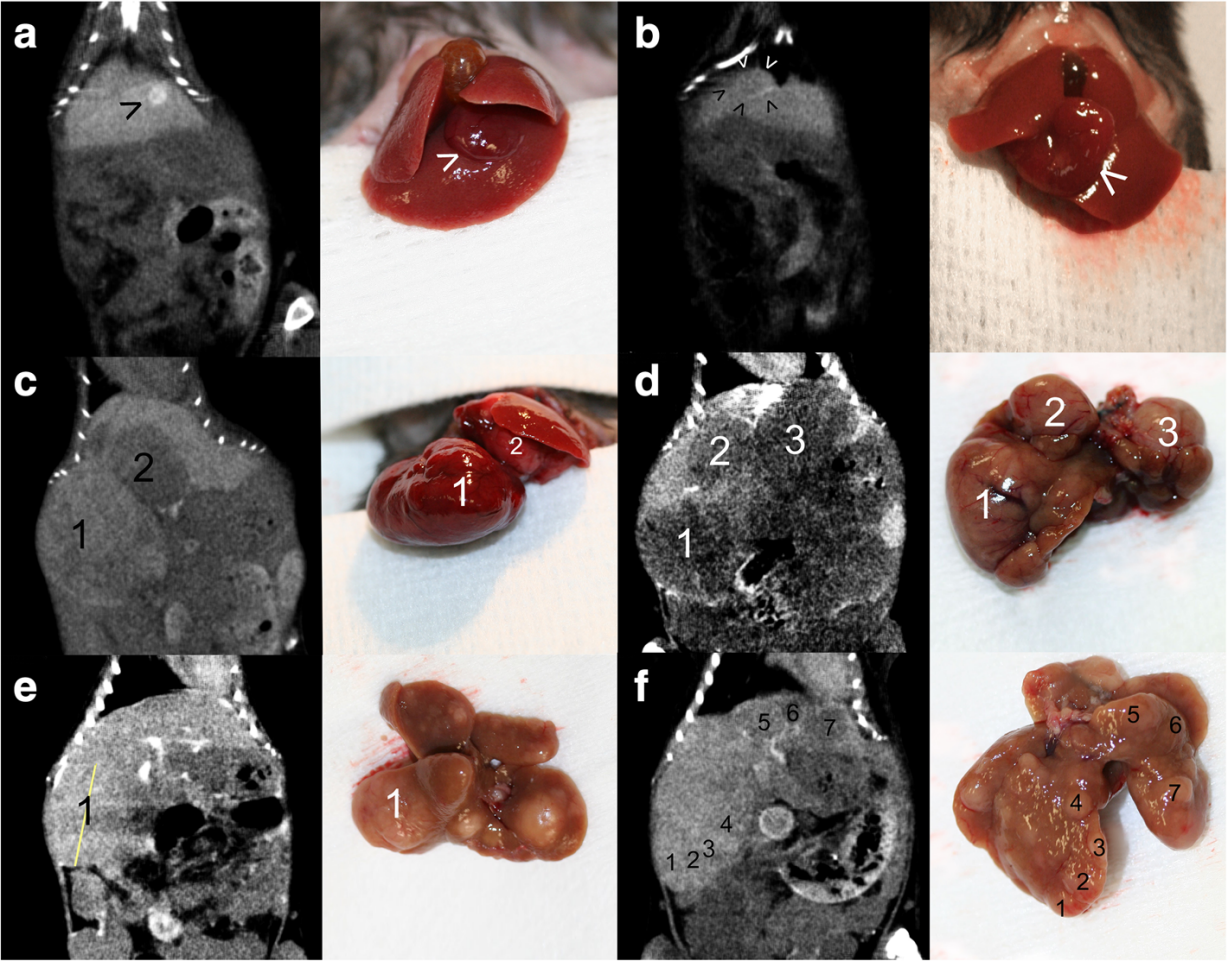
Micro-CT-scan rendering and corresponding gross appearance of hepatocellular carcinomas (HCC) in *Trim24* ^L2/L2^Alb-Cre mice. **a** Hyperdense, hypervascular nodule in the left liver lobe (*arrowhead*). **b** Isodense nodule, with distinguished vascular enhancement ring in the left liver lobe (*arrowheads*). **c** Presence in the right median liver lobe of a hypervascular, isodense nodule [1] and in the left median liver lobe of a hypodense nodule [2]. **d** Multifocal HCC with three well-delineated, hypodense nodules [1–3]. **e** Multifocal HCC with multiple isodense nodules with an average diameter of 3 to 4 mm; only one tumor [1] located in the right liver lobe had been identified by micro-CT-scan imaging (*vertical* diameter shown). **f** Multifocal HCC with multiple hypodense nodules, isodense nodules and isodense nodules with vascular enhancement ring [1–7] – 3 mm in diameter, with edges difficult to evaluate by micro-CT-scan imaging and at dissection

The radiological rendering of liver tumors in *Trim24*^L2/L2^Alb-Cre mice after contrast enhancement showed a hyperdense, isodense or hypodense nodular or diffuse mass (Fig. 2).

*Hyperdense hypervascular tumors* were observed especially during the early development of the disease and corresponded to small tumors (Fig. 2a).

*Isodense areas with a distinguished vascular enhancement ring* (Fig. 2b) were also observed during the early development of the disease and were consistent with tumor diagnosis. Isodense tumors represent a real challenge for diagnosis because there is no difference between normal and tumor tissue in certain views (frontal/sagittal/axial). Important arguments in favor of an isodense tumor are the presence of highly inhomogeneous areas (Fig. 2c, e, and f) as well as the normal shape disruption of the liver lobe. Sometimes, a very small disturbance of the normal anatomy is seen and the presence of some residual peritoneal contrast agent might be beneficial and facilitate this finding, by delineating the tumor’s edges (Fig. 2c and e).

*Hypodense areas* were observed at all stages of tumor development and corresponded to less vascularized tumors (Fig. 2c and d). Hypodense areas might otherwise correspond to central necrosis within larger isodense or hypervascular tumors (Fig. 2c).

**Fig. 3:**
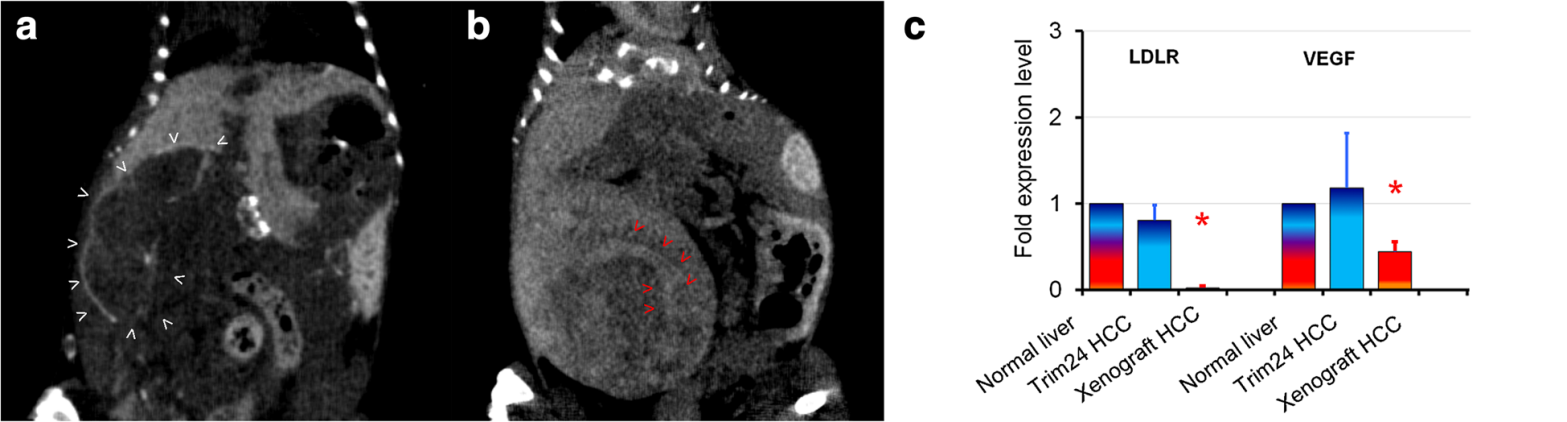
Micro-CT-scan rendering of hepatocellular carcinoma (HCC) in mutant versus xenograft mouse model and corresponding gene expression levels. **a** Large hypodense, hypovascularized HCC in orthotopic Hep 55.1c xenograft model in C57BL/6 J mouse (*white arrowheads*). **b** Large, single isodense tumor in *Trim24* ^L2/L2^Alb-Cre mouse with evident neovascularization network (*red arrowheads*). **c** Gene expression of key markers of lipid uptake capacity (LDLR) and blood network development (VEGF) in HCCs compared to corresponding non-tumor liver expression (fold expression level +/− standard deviation). In mutant *Trim24* ^L2/L2^Alb-Cre mice’ HCCs (n = 5) LDLR expression and VEGF and were not significantly different from non-tumor liver (*p* = 0.071 and respectively *p* = 0.56). In orthotopic Hep 55.1c xenografted HCCs (n = 3), LDLR expression was significantly lower than non-tumor liver (* *p* = 0.0001), as well as VEGF expression (* *p* = 0.015)

Unlike HCCs developed in xenograft models, which are constantly visualized on micro-CT-scan as hypodense, hypovascularized areas (Fig. 3a), the hypodense HCC present in *Trim24*^L2/L2^Alb-Cre mice continue to be well-vascularized, with a blood supply which can be identified when thoroughly examining all CT planes (Fig. 3b).

This difference in image rendering by the contrast-enhanced micro-CT-scan was evaluated using gene expression profiling.

The levels of Low Density Lipoprotein receptor (LDLR), which is responsible for Fenestra™ uptake (mediated by the ApoE receptor), and of VEGF which is responsible for neovascularization were comparable to the non-tumor liver in spontaneously developing tumors (*p* > 0.05) and significantly lower in the xenograft model (*p* = 0.0001 for LDLR and *p* = 0.015 for VEGF) (Fig. 3c).

### Accuracy evaluation of contrast-enhanced micro-CT-scan

#### Malignancy detection

In the 28 mice group, micro-CT diagnosis was accurate in 23 mice. True negative results were obtained on 10 micro-CT-scans. True positive results were obtained on 13 micro-CT-scans.

Two false negative results were recorded for isodense large tumors. No tumor sign could be prospectively identified on the micro-CT rendering (see Additional file 3).

Three false positive results were found in three mice in which the micro-CT-scan showed very small suspect hypodense areas. Corresponding volumes of suspected tumors were less than 1 mm^3^ (i.e., 0.52 mm^3^, 0.30 mm^3^ and 0.94 mm^3^). In these animals, no tumor was found at dissection or in histology.

The accuracy of micro-CT-scan tumor detection was 82.1 %, with a positive predictive value of 81.3 % and a negative predictive value of 83.3 %.

#### Number of tumors and volume evaluation

Findings concerning the number of tumors and total tumor volume were further compared between micro-CT-scan imaging and autopsy (confirmed by histological analysis) in the 13 mice, which were correctly diagnosed with HCC.

**Fig. 4:**
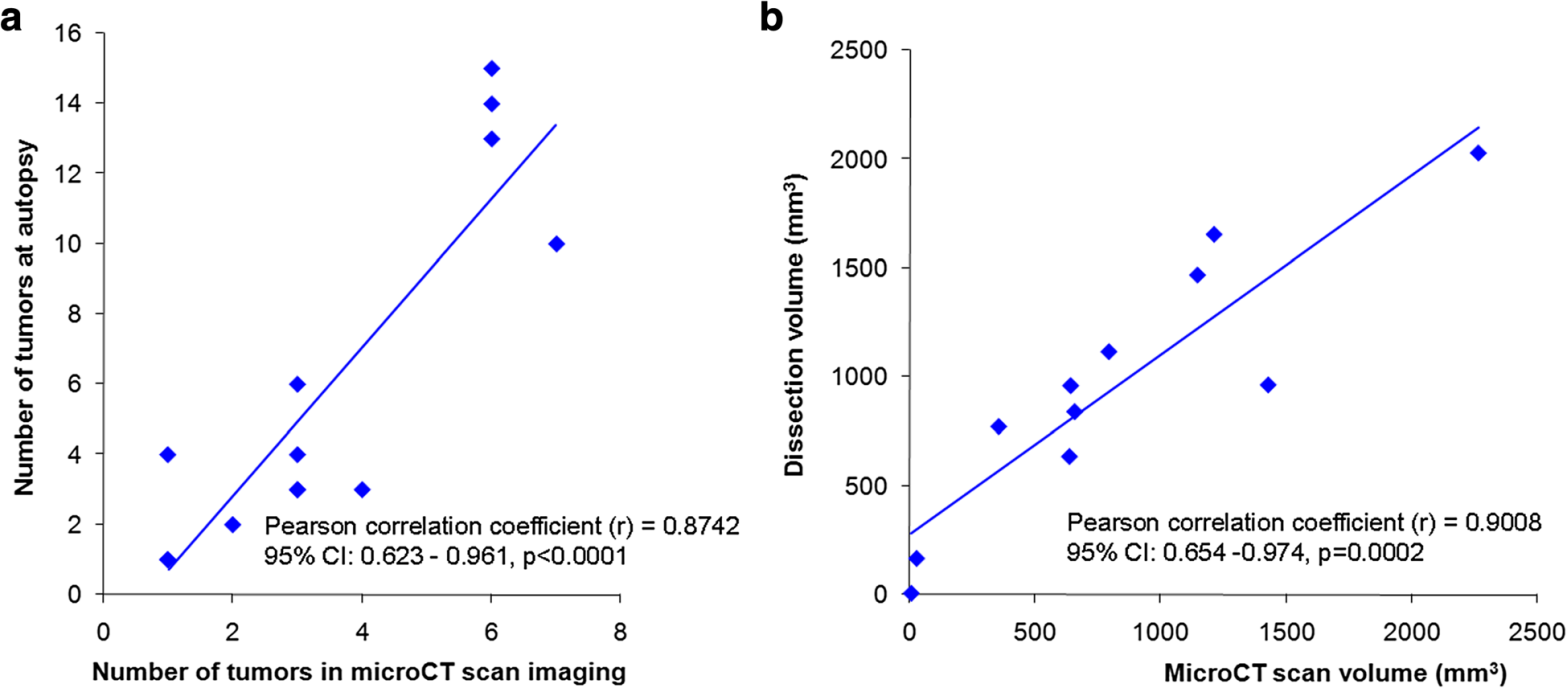
Evaluation of micro-CT-scan accuracy. **a** Correlation between identified tumor number by micro-CT-scan (X axis) and at autopsy (Y axis) follows a linear regression model. The tumor number is accurately predicted only for a small number of tumors (i.e. <3). **b** Correlation between estimated tumor volume by micro-CT-scan (X axis) and at autopsy (Y axis) follows a linear regression model. The regression slope of 0.82 corresponded to a slight underestimation of micro-CT-scan volumes, probably due to physiological organ compression in the living animal

The number of tumors was correctly estimated by micro-CT-scan (Pearson correlation coefficient *r* = 0.874, 95 % CI: 0.623-0.961, *p* < 0.0001) (Fig. 4a).

In one mouse, the two tumors individualized on micro-CT-scan formed a single tumor upon dissection. In seven mice bearing multiple disease, there was a slight underestimation of tumor number. For these mice, tumor volume computation was used to evaluate the extent of the disease. 3D reconstruction of micro-CT-scan rendering and tumor volume computation in a dynamic manner allowed for the most accurate follow-up of tumor mass progression, in both single tumor and multifocal HCC (see Additional file 4).

The average computed tumor volumes measured using micro-CT-scan (832 ± 627 mm^3^) and autopsy (964 ± 523 mm^3^) were equivalent (*p* = 0.15). A highly significant correlation was obtained for tumor volume measurements (Pearson correlation coefficient *r* = 0.90, CI: 0.654 – 0.974, *p* = 0.0002) using micro-CT-scan examination when compared to autopsy findings (Fig. 4b).

### HCC follow-up under ITPP treatment

**Fig. 5:**
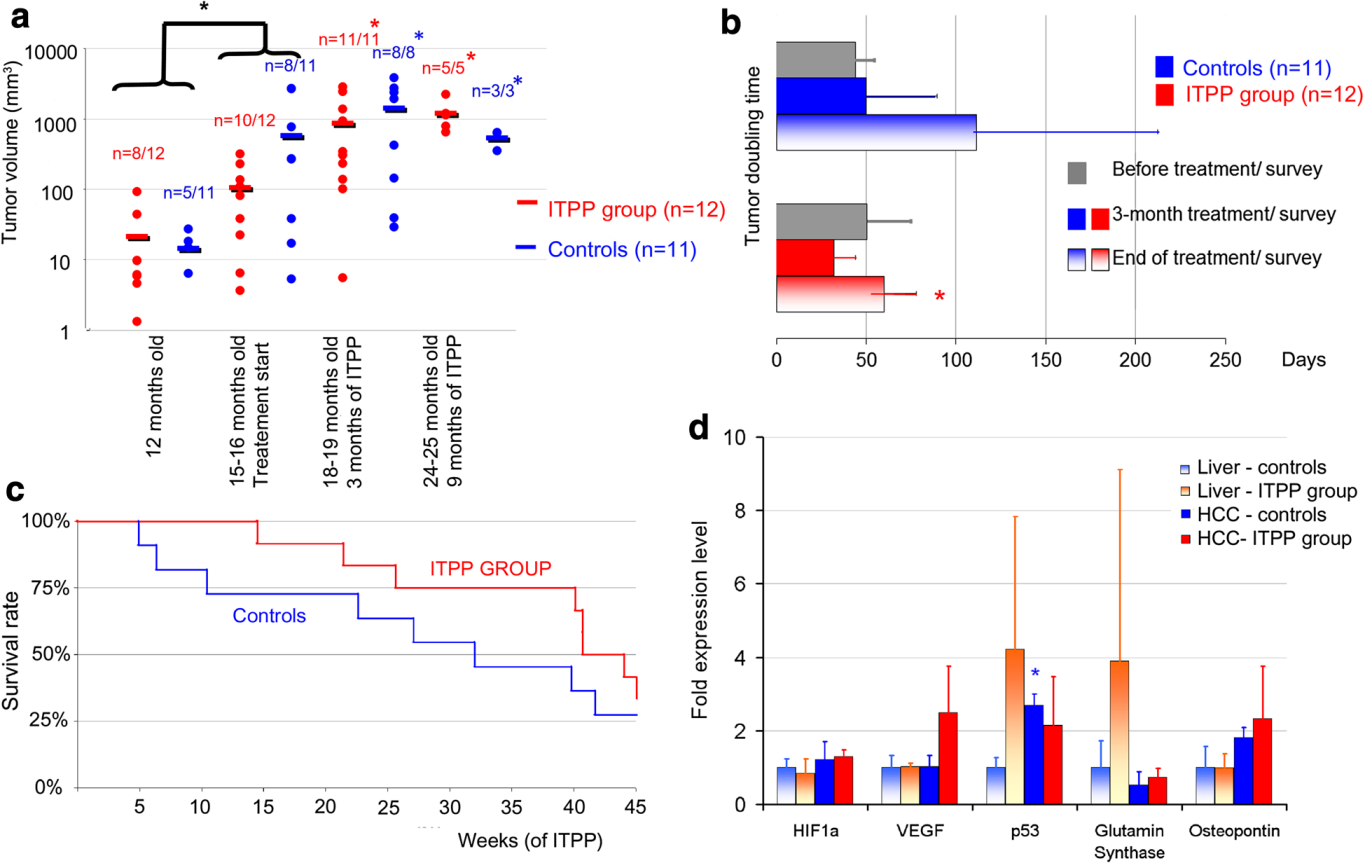
Hepatocellular carcinoma (HCC) follow-up under myo-inositol trispyrophosphate (ITPP) treatment (*red*) versus control group (*blue*) of hepatocyte-specific *Trim24*-null mice. **a** Follow-up of tumor volumes by micro-CT-scan imaging. Logarithmic scale was employed in order to cover the entire volume range. No significant difference was observed between the tumor volumes in the ITPP group and in the control group at any time point. Before treatment started (12 to 15–16 months), there was a significant progression in tumor volume in both groups (*black, *p* = 0.043). At 3 and 9 months after the treatment started (15–16 to 18–19 months), the tumor burden in live animals was in significant progression in the control group (*blue) and in ITPP group (*red). **b** Tumor doubling time (TDT), on the basis of tumor volumes obtained by micro-CT. No difference was observed in TDT between the ITPP group and the control group, at any time point. In the ITPP group, mild tumor growth acceleration was observed at 3 months of treatment with significant deceleration at 9 months (*p* = 0.008). **c** Survival curves. Median survival interval was 296 days in the ITPP group and 223 days in the control group corresponding to a 2-month prolongation of life span. Log-rank comparison demonstrated improved survival in the ITPP group (*p* = 0.0027). **d** Gene expression level in tumor (full bars) and non-tumor livers (motif bars) in sacrificed hepatocyte-specific *Trim24*-null mice, at the end of the follow-up period. Data were expressed as average fold expression +/− standard deviation. In the control group, the p53 expression level was significantly higher in tumor over non-tumor liver (*blue, *p* < 0.05)

In the group of the 23 hepatocyte-specific *Trim24*-null mice, no tumors were detected before 5 months of age. Tumors were detected in 13 mice at 12 months of age (volume of 18 ± 24 mm^3^) with no difference in tumor volume between groups (Fig. 5a).

At 15–16 months, tumors were detected in 18 mice. There was no difference between tumor volume in the control group (524 ± 862 mm^3^) and the ITPP group (106 ± 97 mm^3^, *p* = 0.24). On the merged group of 23 mice, a significant tumor growth was observed at this time-point, as compared to the volumes observed at 12 months of age (*p* = 0.043, Fig. 5a). The treatment/survey was initiated at this time.

#### Tumor volume follow-up

The follow-up at 3 months showed a tumor volume in the control group of 1450 ± 1389 mm^3^ and of 883 ± 934 mm^3^ in the ITPP group (*p* = 0.36, Fig. 5a). During this period, three animals died in the control group and one animal died in the ITPP group. When considering tumor burden in live animals, a significant increase in tumor volume was demonstrated in the control group (from 511.4 ± 869.8 mm^3^ to 1450 ± 1389 mm^3^, *p* = 0.034) and in the ITPP group (96 ± 97.7 mm^3^ to 883 ± 934 mm^3^, *p* = 0.008).

Mean tumor volume was subject to a high variance.

The follow-up at 9 months showed a tumor volume in the control group of 543 ± 134 mm^3^ and of 1214 ± 566 mm^3^ in the ITPP group (*p* = 0.076, Fig. 5a). Five animals bearing large tumors died in the control group and six animals in the ITPP group. When considering tumor burden in live animals, a significant increase in tumor volume was demonstrated in the control group (from 163.3 ± 224 mm^3^ to 543 ± mm^3^, *p* = 0.041) and in the ITPP group (from 179.5 ± 128.2 mm^3^ to 1214 ± 566 mm^3^, *p* = 0.005).

#### Tumor doubling time

No difference was observed in TDT before treatment in the ITPP group (51 +/− 24 days) versus the control group (44 +/− 10.9 days, *p* = 0.28, Fig. 5b).

In the control group, a mild increase in TDT was observed during follow-up (*p* > 0.05) corresponding to a less rapid tumor growth in the later stages of HCC development (Fig. 5b).

In the ITPP group, a mild decrease in TDT was observed at 3 months of treatment (31.9 +/− 12 days, *p* > 0.05) followed by a significant increase in TDT at 9 months (59.8 +/− 18.3 days, *p* = 0.008), with return to pre-treatment values (Fig. 5b).

No significant difference was observed in TDT between the ITPP group and the control group, at any time point.

#### Survival curve

Overall survival of control animals was 658 +/− 111 days (with an average of 205 ± 113 days of controlled survey). Overall survival in the ITPP group was 720 +/− 87 days, with an average of 265 ± 76 days of ITPP treatment, corresponding to a 2-month prolongation of lifespan (Fig. 5c). Log-rank comparison demonstrated an improved survival in the ITPP group (*p* = 0.0027).

#### Molecular findings

Expression levels of marker genes for the main pathways of carcinogenesis and angiogenesis were investigated in tumor and non-tumor livers, at the end of the follow-up.

Hypoxia pathways were not repressed: HIF1α and VEGF gene expression levels in the liver and tumors was not modified in the ITPP group versus the control group (Fig. 5d). VEGF expression level demonstrated a 2.5-fold increase in the treated tumors (*p* = 0.26).

The expression level of p53 was assessed. In the control group, an over-expression of p53 was recorded in tumors (2.7 +/− 0.3) as compared to livers (1 +/− 0.27, *p* = 0.004). In the ITPP group, the expression profile of the p53 expression was inversed, with a higher expression in livers than in tumors (4.23 +/−3.06, versus 2.1 ± 1.3, *p* > 0.05).

Other carcinogenesis and inflammation markers (glutamine synthase, osteopontin) were not significantly modified (Fig. 5d).

## Discussion

The major interest of transgenic mouse models of HCC is that tumor development reflects the clinical reality: pathological liver background, natural tumor - liver interactions, and well-vascularized tumors. This in vivo animal study valorized the genetic knowledge on the *Trim24* gene, which had enabled the engineering of a HCC mouse model - *Trim24*^L2/L2^Alb-Cre. In practice, to test an anticancer agent in a genetically induced model presents reputable disadvantages: long tumor developing time, unpredictability of tumor development, and non-homogeneity of the model. In order to challenge these shortcomings, a step-by-step methodology was described in this study.

First, evidence was provided that PH resulted in prolonged cell-cycling, with significantly high proliferation rate, beyond 15 days after PH and resulting in earlier development of HCC at long term follow-up. Based on this finding, PH was systematically performed in all animals, in order to boost the hepatocarcinogenesis process.

In order to assess the presence and to ensure the follow-up of HCC in *Trim24*^L2/L2^Alb-Cre mice, a contrast-enhanced micro-CT-scan imaging protocol was created. Several issues needed to be addressed: understanding of the particular rendering of the spontaneously developed tumors, coping with repeated contrast agent injection on the pathological liver background and with toxicity of the contrast agent. Data from the literature are mainly based on graft model imaging, in which the high resolution of the micro-CT-scan, along with specifically developed contrast agents [9, 10, 19, 20] allowed to detect liver tumors starting with a 300 μm diameter [9, 10], to calculate in vivo tumor volume [21], and to monitor disease progression [22, 23]. Monitoring of drug/therapy efficacy using micro-CT-scan was shown to be feasible in orthotropic models [24, 25] and in a pilot study in three transgenic ASV-B mice [10]. In these studies, HCCs were visualized as non-enhanced areas on the background of the normal, contrast-enhanced liver parenchyma. These renderings are consistent with our team’s experience in xenograft tumor models (Fig. 3a). However, they were not confirmed for spontaneously developing tumors in *Trim24*^L2/L2^Alb-Cre mice, in which the rendering of HCC was much more polymorphic (i.e., hypodense, isodense or hyperdense nodules and diffuse mass).

In the present study, a contrast agent injection protocol was described, consisting in an intraperitoneal contrast agent injection 6 h before imaging and vascular agent injection 15 min prior to imaging. This protocol allowed for the optimal visualization of spontaneously developing liver tumors, with low toxicity, adapted to the pathological liver background. Diagnosis of liver malignancy was achieved with an accuracy of 82.1 %, comparable to that obtained in prospective clinical trials [26].

Finally, ITPP was pharmacologically tested in *Trim24*^L2/L2^Alb-Cre spontaneous HCC model. ITPP is a synthetic allosteric effector of hemoglobin, which increases the oxygen-releasing capacity of red blood cells leading to the suppression of hypoxia-inducible factors and to the down-regulation of hypoxia-inducible genes. Consequently, tumor growth is markedly affected in hypoxic tumors [12]. ITPP proved its anticancer efficacy in graft models of melanoma and breast cancer [12], colon cancer [13], pancreatic cancer [14], and hepatoma [25]. In the present setting, the non-invasive imaging approach allowed to collect comprehensive data on tumor growth rate, survival and molecular findings with minimal animal sacrifice.

Tumor growth could be objectified before the ITPP treatment was started, and at the 3 and 9 months follow-up. Significant progression of tumor burden was equally demonstrated in the control group as well as in the ITPP group with no difference between groups. However, when further analyzing TDT, a mild acceleration of tumor growth (decrease of TDT) at 3 months of ITPP treatment was observed, with return to initial growth rate at 9 months of follow-up. Tumor growth acceleration seemed to be in contradiction with previous data [25]. It was nevertheless consistent with molecular findings, which could not demonstrate HIF1a and VEGF suppression at the end of the follow-up period. These findings were probably the consequence of the more important vascularization of tumors, as compared to graft models. In this genetically induced model, in which hepatocytes are continuously cell cycling, hypoxia might be of minimum importance in the neoplastic development, and subsequently the efficacy of the treatment on tumor burden is of less importance. In return, in the ITPP group, tumors were better tolerated, mice continuing to survive despite very large tumors and a low volume of functional livers. In the ITPP group, treatment seemed to protect against the onset of new HCCs, as suggested by higher levels of p53 in non-tumor livers. This could account for the long-term effects of the product, with a significant prolongation of survival, which favored the ITPP treatment.

## Conclusion

Double contrast micro-CT scan approach allowed tumor detection and lifelong follow-up of the spontaneously developing HCC in *Trim24*^L2/L2^Alb-Cre mice. Intention-to-treat pharmacological testing of ITPP was performed with a minimum number of mutant animals and resulted in a 2 months prolongation of lifespan in the ITPP group, despite progressive increase in tumor burden in both groups.

## Additional files

Additional file 1: Fenestra™ toxicity. Table: Liver function tests, expressed as average ± standard deviation, in a 20 mice group (16 wild types and 4 mice *Trim24* ^L2/L2^Alb-Cre) receiving a total dose of up to 22 μl/g Fenestra™) controlled by five wild type mice receiving no Fenestra™ ASAT: aspartate transaminase, ALAT: alanine transaminase, GGT: gamma-glutamyl transferase. Alkaline phoshpatase levels were not different. Serological measurements (ASAT, ALAT, bilirubin, GGT, alkaline phoshpatase) were performed on blood samples collected by direct cardiac puncture under general isoflurane anaesthesia before animal euthanasia. Standard techniques (ADVIA 2400, Siemens and immune-nephelometry BNII) were employed. Figure: Micrographs of histological structure of the liver (A, B, C) and kidney (D) in mice with acute toxicity after 22 μl/g Fenestra™ administration. HE staining, original magnifications x1500 (A, B) and x240 (C, D). Red arrows: leucocyte adhesion and diapedesis; Arrowheads: hepatocyte necrosis; White arrows: periportal or centrolobular inflammatory infiltrate; Black arrows: acute tubular necrosis. (DOC 8191 kb) Additional file 2: Gene primers. A. Primers used for *Trim24* genotyping. B. Primers used for qPCR. (DOC 34 kb) Additional file 3: False negative results in microCT scan imaging in two mice. A, B, C. Aspects corresponding to the first animal. A, B. Axial and frontal view of microCT scan imaging of an isodense, poorly delineated right liver lobe tumor. Prospective and retrospective interpretation of images could only identify an enlargement of the right liver lobe. The border between normal and pathological tissue could not be identified on the microCT scan rendering. C. Gross appearance of the poorly delineated right liver lobe tumor, depicted in A and B. D, E, F. Aspects corresponding to the second animal. D. Retrospective interpretation of microCT scan rendering with identification of a centroabdominal in-homogeneous, isodense tumor (arrowheads). E. Gross appearance of the centroabdominal tumor which was not identified at the prospective interpretation of the microCT scan images. F. Histological assessment of the presence of a hepatocellular carcinoma (HE, x240). (JPG 3580 kb) Additional file 4: Follow-up of hepatocellular carcinoma (HCC) growth by 3D reconstruction. Follow-up of hepatocellular carcinoma (HCC) growth by 3D reconstruction of micro-CT rendering. Micro-CT imaging was performed at 12, 15, and 21 months of age respectively in hepatocyte specific *Trim24*-null mice. A, B, C Exponential growth of unifocal HCC. A. Micro-CT-scan rendering in coronal slides (strips indicate vertical diameter of HCC). B. 3D reconstruction of the micro-CT-scan (HCC is depicted in green). C. Macroscopic appearance of the liver and HCC at dissection at 22 months old (black arrow indicates tumor). D, E, F. Multifocal growth of HCC. D. Micro-CT scan rendering in coronal slides (strips indicate vertical diameters of the HCCs). E. 3D reconstruction of the micro-CT-scan (HCCs are depicted in green). F. Macroscopic appearance of the liver and HCCs at dissection at 23 months old (black arrows indicate tumors). (JPG 4259 kb)

## Abbreviations

HCC: Hepatocellular carcinoma
ITPP: Myo-inositol trispyrophosphate
Micro-CT: High-resolution micro-computed tomography
TDT: Tumor doubling time
*Trim24*: Tripartite motif protein 24
