# Supplementary figures and images for: Development of a methodology for in vivo follow-up of hepatocellular carcinoma in hepatocyte specific Trim24-null mice treated with myo-inositol trispyrophosphate
Source: J Exp Clin Cancer Res. 2016 Sep 29;35:155. doi: 10.1186/s13046-016-0434-8 (PMC5041534; doi:10.1186/s13046-016-0434-8)

|  | ASAT (U/l) | ALAT (U/l) | Bilirubin (µmol/l) | GGT (U/l) |
| --- | --- | --- | --- | --- |
| FenestraTM group (20 mice) | 506±523 | 120±122 | 1.2±2 | <5 |
| Control group (5 mice) | 194±89 | 42±14 | 0±0 | <5 |
| p | 0.046 | 0.019 | 0.023 | / |


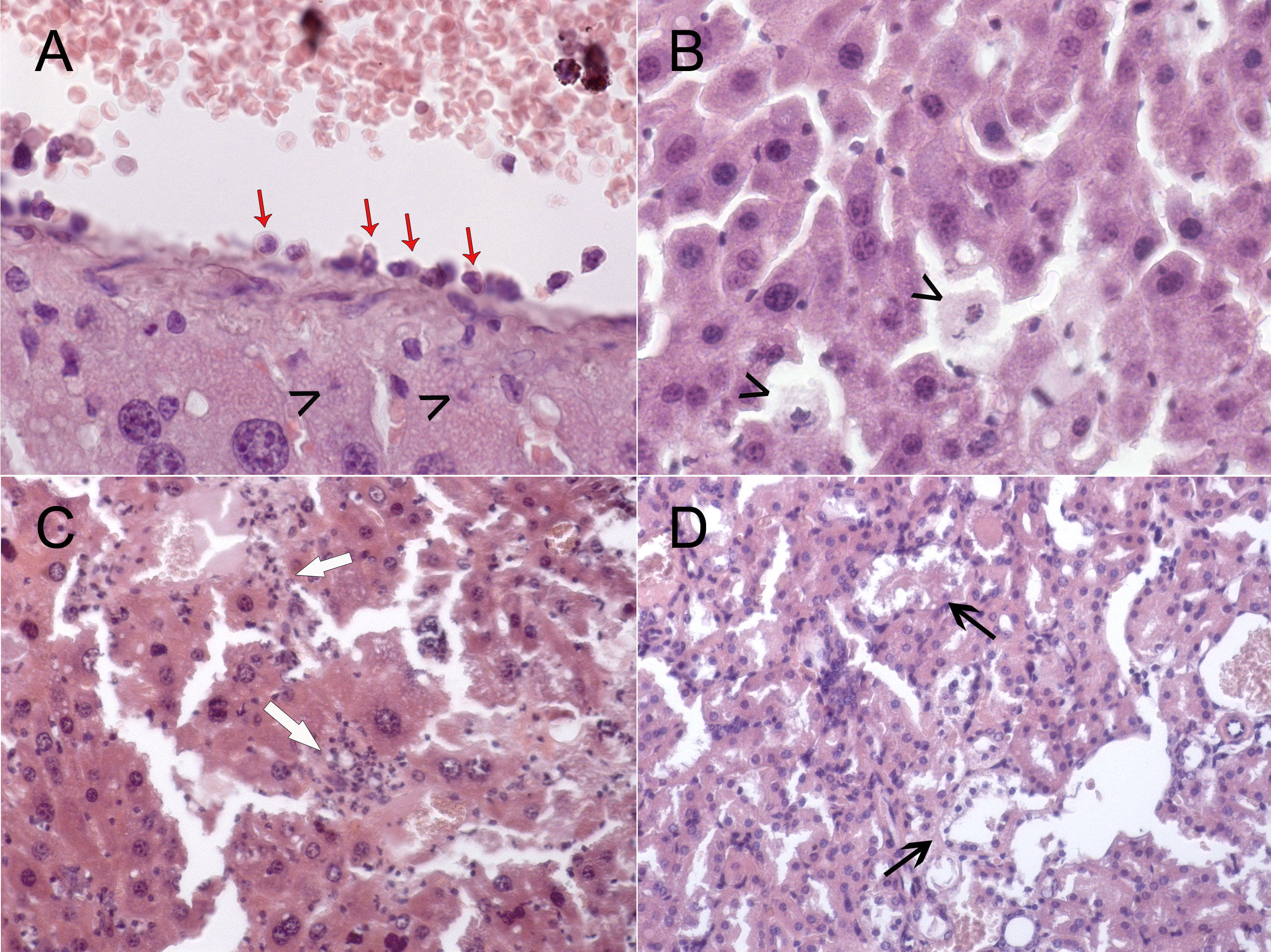

Supplement: Additional file 1: — Fenestra™ toxicity. Table: Liver function tests, expressed as average ± standard deviation, in a 20 mice group (16 wild types and 4 mice Trim24 L2/L2Alb-Cre) receiving a total dose of up to 22 μl/g Fenestra™) controlled by five wild type mice receiving no Fenestra™ ASAT: aspartate transaminase, ALAT: alanine transaminase, GGT: gamma-glutamyl transferase. Alkaline phoshpatase levels were not different. Serological measurements (ASAT, ALAT, bilirubin, GGT, alkaline phoshpatase) were performed on blood samples collected by direct cardiac puncture under general isoflurane anaesthesia before animal euthanasia. Standard techniques (ADVIA 2400, Siemens and immune-nephelometry BNII) were employed. Figure: Micrographs of histological structure of the liver (A, B, C) and kidney (D) in mice with acute toxicity after 22 μl/g Fenestra™ administration. HE staining, original magnifications x1500 (A, B) and x240 (C, D). Red arrows: leucocyte adhesion and diapedesis; Arrowheads: hepatocyte necrosis; White arrows: periportal or centrolobular inflammatory infiltrate; Black arrows: acute tubular necrosis. (DOC 8191 kb) [file 13046_2016_434_MOESM1_ESM.doc]

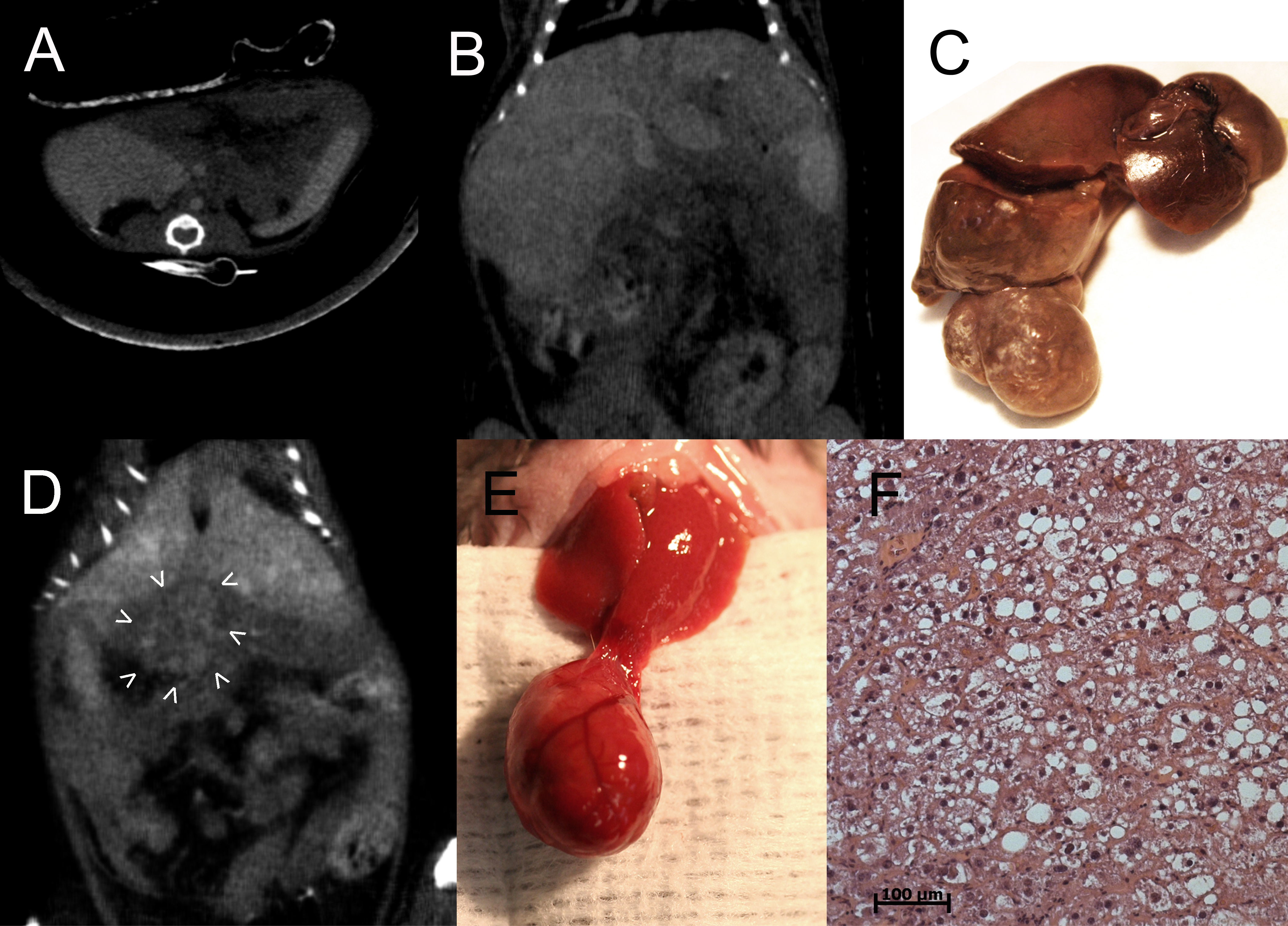

Supplement: Additional file 3: — False negative results in microCT scan imaging in two mice. A, B, C. Aspects corresponding to the first animal. A, B. Axial and frontal view of microCT scan imaging of an isodense, poorly delineated right liver lobe tumor. Prospective and retrospective interpretation of images could only identify an enlargement of the right liver lobe. The border between normal and pathological tissue could not be identified on the microCT scan rendering. C. Gross appearance of the poorly delineated right liver lobe tumor, depicted in A and B. D, E, F. Aspects corresponding to the second animal. D. Retrospective interpretation of microCT scan rendering with identification of a centroabdominal in-homogeneous, isodense tumor (arrowheads). E. Gross appearance of the centroabdominal tumor which was not identified at the prospective interpretation of the microCT scan images. F. Histological assessment of the presence of a hepatocellular carcinoma (HE, x240). (JPG 3580 kb) [file 13046_2016_434_MOESM3_ESM.jpg]

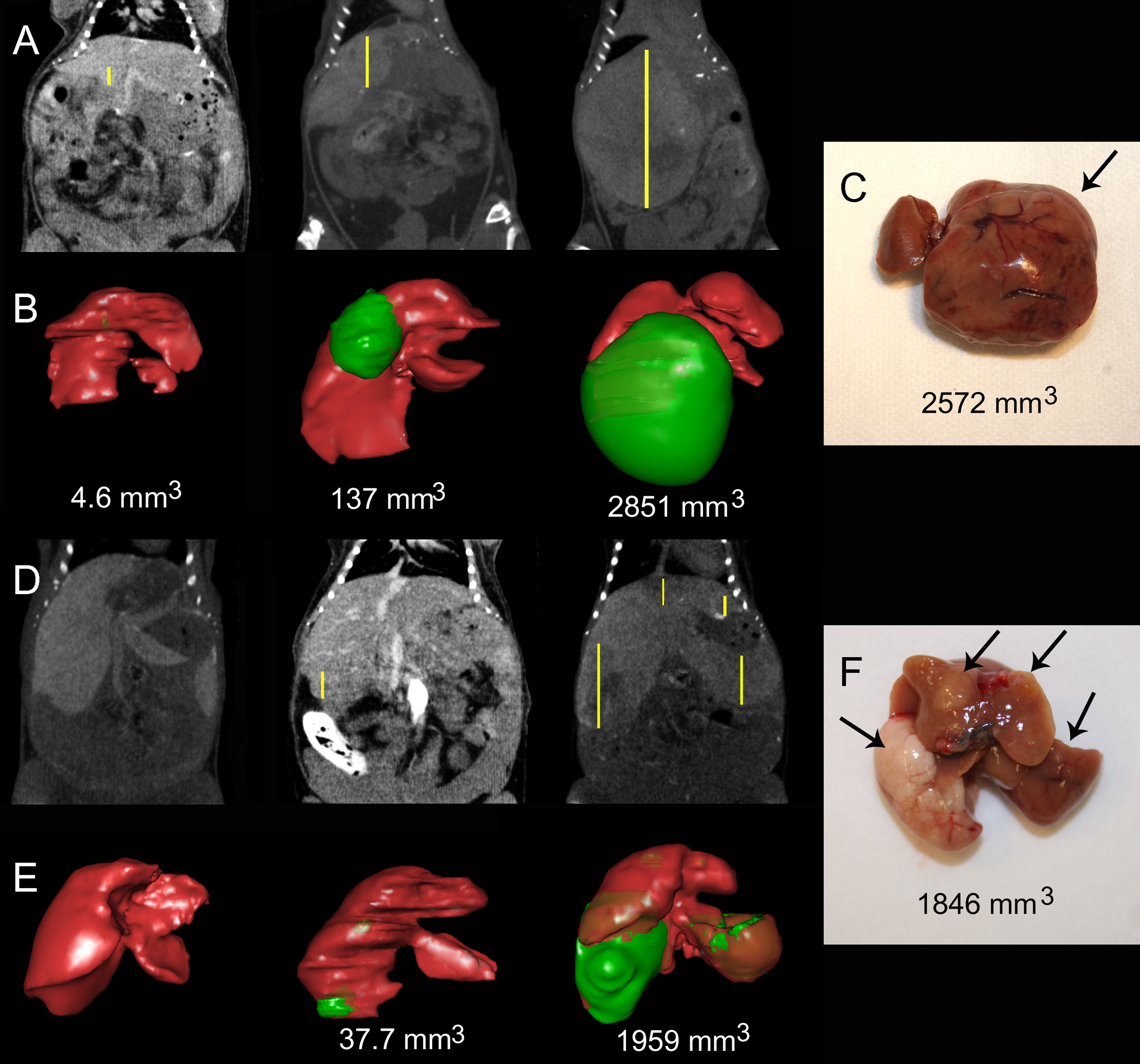

Supplement: Additional file 4: — Follow-up of hepatocellular carcinoma (HCC) growth by 3D reconstruction. Follow-up of hepatocellular carcinoma (HCC) growth by 3D reconstruction of micro-CT rendering. Micro-CT imaging was performed at 12, 15, and 21 months of age respectively in hepatocyte specific Trim24-null mice. A, B, C Exponential growth of unifocal HCC. A. Micro-CT-scan rendering in coronal slides (strips indicate vertical diameter of HCC). B. 3D reconstruction of the micro-CT-scan (HCC is depicted in green). C. Macroscopic appearance of the liver and HCC at dissection at 22 months old (black arrow indicates tumor). D, E, F. Multifocal growth of HCC. D. Micro-CT scan rendering in coronal slides (strips indicate vertical diameters of the HCCs). E. 3D reconstruction of the micro-CT-scan (HCCs are depicted in green). F. Macroscopic appearance of the liver and HCCs at dissection at 23 months old (black arrows indicate tumors). (JPG 4259 kb) [file 13046_2016_434_MOESM4_ESM.jpg]
