# Supplementary material for: Development of a methodology for in vivo follow-up of hepatocellular carcinoma in hepatocyte specific Trim24-null mice treated with myo-inositol trispyrophosphate
Source: J Exp Clin Cancer Res. 2016 Sep 29;35:155. doi: 10.1186/s13046-016-0434-8 (PMC5041534; doi:10.1186/s13046-016-0434-8)
Supplement: Additional file 2: — Gene primers. A. Primers used for Trim24 genotyping. B. Primers used for qPCR. (DOC 34 kb) [file 13046_2016_434_MOESM2_ESM.doc]

**Additional file 2**.

A. Primers used for *Trim24* genotyping

| Name | Sequence | Gene |
| --- | --- | --- |
| UC217 | TGTGGGAGTTGGTTCTCCTG | *Trim24* |
| ABG187 | AGACAGGCAGCAGCAGTTAC | *Trim24* |
| ABG188 | GGC CAG TGA GAT ACT TCA GC | *Trim24* |
| TK139 | ATT TGC CTG CAT TAC CGG TC | *Cre* |
| TK141 | ATC AAC GTT TTG TTT TCG GA | *Cre* |

B. Primers used for qPCR

| Gene | Forward primer | Reverse primer |
| --- | --- | --- |
| *LDLR* | CAGTGTCCCACCAAGTCCA | GTTCTTCAGCCGCCAGTTC |
| *VEGF* | GGACCCTGGCTTTACTGCT | GGTGATGTTGCTCTCTGACG |
| *HIF1*α | ACGACCACTGCTAAGGCATC | TGCTCCGTTCCATTCTGTTC |
| *p53* | GCTGGATAGGAAAGAGCACA | GTTGAGGGCAAGAAATGGAG |
| Glutamin synthase | TAACCCGCCTCAACTTTCTG | AATCACCCTCGCAGTCCTCT |
| Osteopontin | GCTTGGCTTATGGACTGAGG | CTCTCCTGGCTCTCTTTGGA |
| *HPRT* | TGTTGTTGGATATGCCCTTG | GGCCACAGGACTAGAACACC |
